# An Overview of Complete Blood Count Sample Rejection Rates in a Clinical Hematology Laboratory Due to Various Preanalytical Errors

**DOI:** 10.7759/cureus.34444

**Published:** 2023-01-31

**Authors:** Tayyab Noor, Ayisha Imran, Hassan Raza, Shereen Umer, Nomaan Aslam Malik, Akhtar Sohail Chughtai

**Affiliations:** 1 Haematology, Chughtai Lab, Lahore, PAK; 2 Hematopathology, Chughtai Lab, Lahore, PAK; 3 Histopathology, Chughtai Lab, Lahore, PAK

**Keywords:** frequency, sample rejection, pre-analytical errors, hematology, complete blood count

## Abstract

Introduction

The Chughtai Laboratory collects blood samples for complete blood counts from various hospitals, emergency departments, ICUs, and through home sampling services all across the country. The preanalytical phase is an integral component of laboratory medicine. A laboratory report has a key role in patient treatment and the clinician's decision in the management of the disease. Preanalytical errors are most frequently caused by the absence of a sample and/or inappropriate understanding of a test request, mislabeling, contamination from the sampling site, hemolyzed, clotted, insufficient samples, storage issues, and inappropriate blood to anticoagulant proportion or inappropriate choice of anticoagulant.

Objective

To identify the cause of rejection rates of the complete blood count samples and reduce the rejection rates by improving the accuracy of the results and lowering pre-analytical errors.

Methods

This cross-sectional study was done in the Hematology Department of Chughtai Laboratory's head office in Lahore between 19-06-2021 and 19-10-2021. Simple random sampling was applied to collect the data. About 3 ml of each blood sample was received in an ethylenediaminetetraacetic acid (EDTA) vial, inspected visually, run on Sysmex XN-9000 (Sysmex Corporation, Kobe, Hyogo, Japan), and was reviewed on peripheral smears.

Results

Out of 231,008 blood samples, 11,897 (5.15%) samples were rejected. The most common pre-analytical mistake was storage issues due to transportation delay (19.45%) followed by wrong medical records (19.16%), diluted samples (16.35%), incorrect tubes (16.01%), hemolyzed samples (15.13%), unlabeled samples (10.01%), and clotted sample (3.88%).

Conclusion

In the hematology department, the total rejection rate observed during the study period was 5.15%. Recognition of preanalytical errors and avoiding them will help us lower the sample rejection rate and raise the overall quality of laboratory management.

## Introduction

Medical diagnostics must be of high quality in order to provide patients with reliable health care. Laboratory medicine, among other clinical fields, plays a critical role in patient safety [1]. Laboratory practice is traditionally divided into three phases: pre-analytical, analytical, and post-analytical. The pre-analytical phase includes the proper sample selection, patient information, sample collection and tagging, sample processing, sorting out, titrations, and centrifugation [2-3].

Any of these steps may be omitted, resulting in inaccurate results that are ascribed to the pre-analytical phase. Despite the fact that each of the three phases should be given consideration on its own to boost laboratory standards, they are all crucial for overall quality control. The most error-prone phase of the method is the pre-analytical phase. Pre-analytical problems are among the most significant obstacles that laboratory professionals have encountered in the previous two decades [4].

The overall inaccurate frequency in lab work, according to Lippi and coworkers, varies from 0.1% to 3.0% [5]. Pre-analytical errors are thought to account for 46% to 68.2% of misdiagnoses while analytical errors, which have been the focus of earlier studies, only make up about 10% of all misdiagnoses [6]. Furthermore, pre-analytical errors account for 18.5% to 47% of all laboratory errors. The most common pre-analytical errors are missing medical information, inadequate containers, and lost samples [7].

Although there are international standards for blood sampling and standardization of the testing process, compliance with guidelines is extremely poor, notably in the past when laboratory personnel were not involved and nurses or resident doctors used to perform the sampling, the rate of preanalytical errors was very high [8]. Besides that, the sample rejection criteria differ from one laboratory to the next [9]. There is a lack of professional data on reporting, root cause analysis, and laboratory error prevention strategies [10]. The aim of this research is to observe the frequency of complete blood count (CBC) sample rejection factors to improve the quality of results and reduce pre-analytical errors. These sample errors are leading to repeated patient collections and testing. The ability to identify problem areas and continuously train phlebotomy personnel are crucial to lowering these errors. Rejecting inappropriate samples may cause a delay in turnaround time and have an impact on patient care. This would help us determine the extent of rejection and reduce the cost of applying testing machines and markers due to a decrease in samples with analytical errors by rejecting them on presentation or initial testing.

## Materials and methods

This cross-sectional study was done in the hematology department of Chughtai Laboratory's head office in Lahore between 19-06-2021 and 19-10- 2021. Simple random sampling was applied to collect the data. All human blood samples for testing of complete blood count were selected. It was a time-bound study, and 231,008 blood samples that were received during the study period were selected for the study. About 3 ml of each blood sample was received in an ethylenediaminetetraacetic acid (EDTA) vial (lavender top K2 EDTA anticoagulant), correct specimen volume, i.e., all EDTA tubes are optimized at 1.5mg.EDTA/ml of whole blood. The minimum amount suggested by BD, the tube vendor is at least a 90% draw volume. For example, the 6.0 ml of EDTA tubes should have at least 5.4 ml of whole blood and the 3.0 ml EDTA tubes should have at least 2.7 ml of whole blood. The following predetermined rejection criteria were used to determine which samples would be rejected: diluted samples, hemolyzed samples, lipemic samples, anticoagulated samples (EDTA) with clots, improper sample transport/storage, improper container closure, specimens delayed in transit invalidating results, and unlabeled, mislabeled, or wrong specimen containers. It was then run on Sysmex XN-9000 (Sysmex Corporation, Kobe, Hyogo, Japan) and subsequently reviewed on peripheral smears by hematologists. The following predetermined rejection criteria were used to determine which samples would be rejected: diluted samples, hemolyzed samples, anticoagulated samples (EDTA) with clots, improper sample transport, improper container closure, specimens delayed in transit invalidating results, and unlabeled specimen containers. The data generated were inspected and analyzed monthly by a trained pathologist. The number of rejected samples and the reason for rejection were recorded on a proforma each month. The data collected in the study period were entered into the computer using a Microsoft Excel sheet (Microsoft Corporation, Redmond, WA). Version 26 of Statistical Package for the Social Sciences (SPSS v 26; IBM Corp., Armonk, NY) was used to analyze the data. Various causes of rejection were evaluated, and the frequency of these preanalytical errors was determined.

Ethical approval was obtained from the Department of Hematology, Chughtai Institute of Pathology. Informed written permission was obtained from the Chughtai Institute of Pathology Institutional Review Board (IRB) and submitted to the head of the institution.

## Results

**Figure 1:**
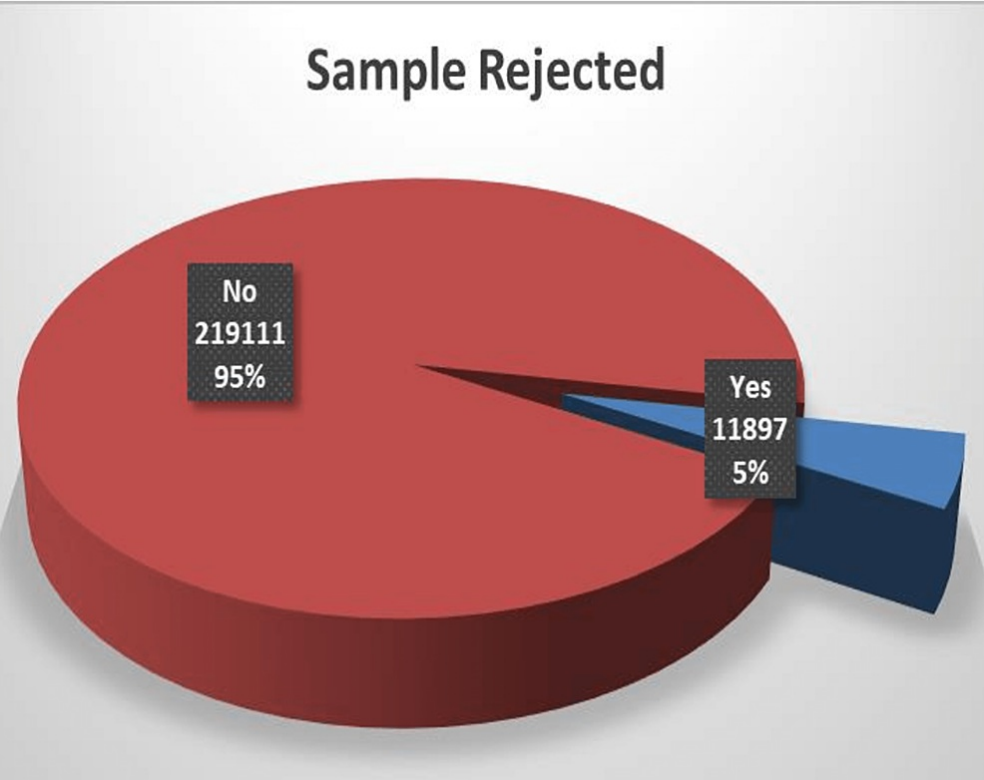
Frequency of pre-analytical errors in CBC samples CBC: complete blood count

The total number of complete blood count samples received during the study period was 231,008 (n=231,008). Out of these, 11,897 (5.15%) samples were rejected at the pre-analytical stage due to some errors (Figure 1).

The most common pre-analytical error was storage changes due to transportation delay, accounting for 2314 rejected samples (19.45%), followed by 2280 rejected samples due to wrong medical records (19.16%). The total number of rejected diluted samples was 1945 (16.35%), and the number of rejected samples due to incorrect tubes was 1905 (16.01%). Hemolyzed samples rejected were 1800 (15.13%). Unlabeled samples rejected were 1191 (10.01%) and the clotted samples constituted 462 (3.88%) of the total rejected samples (Table 1).

**Table 1:** Types of errors that lead to sample rejection

Type of errors	Frequency	Percentage
Storage changes	2314	19.45
Wrong medical record	2280	19.16
Diluted samples	1945	16.35
Incorrect tubes	1905	16.01
Sample hemolyzed	1800	15.13
Unlabeled samples	1191	10.01
Clotted samples	462	3.88

## Discussion

Our findings are consistent with research done in other developing countries [11]. A five-year Spanish study found that pre-analytical errors occurred at a rate of 0.047% overall [12]. The variable with the highest frequency in previous studies was the “hemolyzed” sample [13]. In this study, the most frequent pre-analytical error found is "sample storage" as shown in Table 1. Negligence in this area can result in a patient's diagnosis being delayed, requiring additional laboratory testing, or being treated for the wrong medical condition. An example of such an error is the incorrect labeling of the EDTA/serum evacuated vials in which the samples are transferred for blood categorization and cross-matching, which led to an acute hemolytic reaction following incompatible blood transfusion. Modern laboratory practice is involved in more than just "reporting of a specimen," as it also involves delivering this critical information to clinicians, so they can diagnose and treat the patients accordingly. Errors in labeling can cause this procedure to be delayed and ineffective.

The next significant mistake in our setup after "labeling" was "diluted" samples (Table 1). The majority of the samples in our laboratory's workload came from inpatient departments, where nursing personnel and junior doctors handle the sampling procedure. They frequently forget how crucial it is to take a blood sample directly from a vein, without the aid of intravenous needles. If a patient's arm is being injected with intravenous fluids, the opposite arm should be used to collect the sample [14]. If an intravenous cannula is inserted into both arms, blood can be drawn after the intravenous infusion has been turned off for a minimum of two minutes and a tourniquet applied below the infusion site.

Approximately 14% of the mistakes in the current investigation were attributed to anticoagulated samples with clogs (EDTA and sodium citrate) (Table 1). Visual inspection of the sample can easily detect gross clots, but micro clots are more challenging to find. The high blood-to-anticoagulant ratio and improper blood-anticoagulant mixing after the anticoagulant is delivered in the tube are the two factors that lead to clots [15]. The overfilling of the EDTA/citrated vials in this study may also be responsible for this inaccuracy.

Our study also revealed that the majority of the samples were diluted in October. When we investigated the fundamental reason for this problem, we noticed that a tertiary care hospital from where we were receiving a large number of EDTA samples reported their complete blood counts reports. New trainee nursing personnel had been hired in October. Therefore, the lack of adequate training and subpar technical can be used to explain this issue. We asked the hospital management to conduct internal training sessions for their nursing staff to educate them on proper phlebotomy methods in order to tackle this issue. Pre-analytical errors in future months fell to an average that was equivalent to the prior months after the training session.

According to numerous research, one of the cornerstones of treatments needed to lower errors at the preanalytical stage is training. In all of these experiments, the error rate considerably decreased after training. The ability of trainees to apply the knowledge and skills they have acquired to their daily work is crucial to the success of training programs. However, research examining how employees perform following training has indicated that trainees instantly apply around 40% of the program's advantages to their work. The occurrence rate of preanalytical errors, however, drops to 25% after six months and to 15% after a year. Regular staff retraining is necessary to prevent this decline in production and the repetition of errors [16].

Low patient satisfaction is directly correlated with laboratory mistakes and high costs for both patients and the lab services system. The negative effects of laboratory errors on patient care extend beyond the fact that they increase turnaround time, need extra redraws, and result in inaccurate diagnoses and unsuitable medications; they also harm a laboratory's reputation and erode patient trust in diagnostic services. It has been estimated that laboratory error has a negative effect on patient outcomes of up to 24.4% [17].

This process is resulting in a financial burden on the health care system on a larger scale. According to a Green study, pre-analytical mistake expenses account for 0.23% to 1.2% percent of the entire operating budget for hospitals [18]. Hospitals in North America reported outpatient expenses of $337.05 (which includes patients from the emergency room), inpatient costs of $162.18 (for critical patients), and inpatient costs of $357.15 (for other patients) [19]. We don't have complete local data on the impact of pre-analytical errors on hospital budgets, but we can be sure that these errors have made things more difficult.

Although there has been a tremendous improvement in laboratory workflow, the pre-analytical phase of laboratory testing still has the highest risk due to the numerous processes that must be taken both before and after the specimen is delivered to the lab. The aforementioned considerations make it clear that using the improper sampling technique is what causes the majority of pre-analytical mistakes [20-21].

In the majority of developed regions, phlebotomy is regarded as a separate branch. We may use a similar strategy to raise the quality of our laboratory work [22-23]. This entire process involves a comprehensive strategy, which includes effective communication between the members of the sample management team, ordering doctors, phlebotomists, delivery companies that deliver the sample, as well as laboratory employees that analyze the substance for testing. Additionally, labs must keep detailed documentation of all errors made during the pre-analytical phase. Consistent adherence to the remedial measures can help to steadily lower error rates [24].

## Conclusions

In the hematology department, the total rejection rate observed during the study period was 5.15%, the major cause of sample rejection being storage changes. To reduce this cause of rejection, transportation shall be done under proper storage conditions with as minimum transit time as possible. Other pre-analytical errors can be reduced by proper training of healthcare staff with periodic assessments. A monitoring system should be devised to observe strict compliance with standard operating procedures. In addition, better coordination with clinicians can minimize preanalytical errors related to misidentification, mislabeling, or wrong vials. Continual medical education sessions shall be conducted for phlebotomists to lower the errors like clots in the vial, hemolyzed samples, insufficient quantity, inappropriate vials, and mislabeling. Order of draw should be followed. The introduction of laboratory automation brings about standardization of workflow and helps get rid of many steps undertaken by humans, which are prone to errors. Automation and advanced robotics have tremendously lowered the chance of preanalytical errors from human factors (stress, fatigue, and cognitive impairment). Close control should only be achieved due to effective assimilation between automation and information management. In particular, while automation initially optimizes the sample assessment, optimizing the processing pathway and scheduling and precise measurements, information management involves reach processes, sample tracking, data entry and reliable reporting, quality control assessment, and documentation. At length, there is always a massive space for improvement in the pre-analytical phase for more effective measurements for reasonable quality control.
